# Risk factors for campylobacteriosis in Australia: outcomes of a 2018–2019 case–control study

**DOI:** 10.1186/s12879-022-07553-6

**Published:** 2022-06-30

**Authors:** Danielle M. Cribb, Liana Varrone, Rhiannon L. Wallace, Angus T. McLure, James J. Smith, Russell J. Stafford, Dieter M. Bulach, Linda A. Selvey, Simon M. Firestone, Nigel P. French, Mary Valcanis, Emily J. Fearnley, Timothy S. Sloan-Gardner, Trudy Graham, Kathryn Glass, Martyn D. Kirk

**Affiliations:** 1 National Centre for Epidemiology and Population Health, The Australian National University, Canberra, ACT Australia; 2 Department of Health, Government of Western Australia, Perth, WA Australia; 3 Agriculture and Agri-Food Canada, Agassiz Research and Development Centre, Agassiz, BC Canada; 4 Food Safety Standards and Regulation, Health Protection Branch, Queensland Health, Brisbane, Qld Australia; 5 School of Biology and Environmental Science, Faculty of Science, Queensland University of Technology, Brisbane, Qld Australia; 6 OzFoodNet, Communicable Diseases Branch, Queensland Health, Brisbane, Qld Australia; 7 Melbourne Bioinformatics, The University of Melbourne, Melbourne, Vic Australia; 8 Microbiological Diagnostic Unit Public Health Laboratory, The Peter Doherty Institute for Infection and Immunity, The University of Melbourne, Melbourne, Vic Australia; 9 Faculty of Medicine, The University of Queensland, Brisbane, Qld Australia; 10 Melbourne Veterinary School, Faculty of Veterinary and Agricultural Sciences, The University of Melbourne, Parkville, Vic Australia; 11 Infectious Disease Research Centre, Massey University, Palmerston North, New Zealand; 12 OzFoodNet, Government of South Australia, Department for Health and Wellbeing, Adelaide, SK Australia; 13 OzFoodNet, Health Protection Service, ACT Health, Canberra, ACT Australia; 14 Public Health Microbiology, Forensic and Scientific Services, Queensland Health, Brisbane, Qld Australia

**Keywords:** Australia, *Campylobacter*, Campylobacter Infections, Case–Control Study, Chickens, Dogs, Foodborne diseases, Proton Pump Inhibitors, Risk Factors

## Abstract

**Background:**

We aimed to identify risk factors for sporadic campylobacteriosis in Australia, and to compare these for *Campylobacter jejuni* and *Campylobacter coli* infections.

**Methods:**

In a multi-jurisdictional case–control study, we recruited culture-confirmed cases of campylobacteriosis reported to state and territory health departments from February 2018 through October 2019. We recruited controls from notified influenza cases in the previous 12 months that were frequency matched to cases by age group, sex, and location. *Campylobacter* isolates were confirmed to species level by public health laboratories using molecular methods. We conducted backward stepwise multivariable logistic regression to identify significant risk factors.

**Results:**

We recruited 571 cases of campylobacteriosis (422 *C. jejuni* and 84 *C. coli*) and 586 controls. Important risk factors for campylobacteriosis included eating undercooked chicken (adjusted odds ratio [aOR] 70, 95% CI 13–1296) or cooked chicken (aOR 1.7, 95% CI 1.1–2.8), owning a pet dog aged < 6 months (aOR 6.4, 95% CI 3.4–12), and the regular use of proton-pump inhibitors in the 4 weeks prior to illness (aOR 2.8, 95% CI 1.9–4.3). Risk factors remained similar when analysed specifically for *C. jejuni* infection*.* Unique risks for *C. coli* infection included eating chicken pâté (aOR 6.1, 95% CI 1.5–25) and delicatessen meats (aOR 1.8, 95% CI 1.0–3.3). Eating any chicken carried a high population attributable fraction for campylobacteriosis of 42% (95% CI 13–68), while the attributable fraction for proton-pump inhibitors was 13% (95% CI 8.3–18) and owning a pet dog aged < 6 months was 9.6% (95% CI 6.5–13). The population attributable fractions for these variables were similar when analysed by campylobacter species. Eating delicatessen meats was attributed to 31% (95% CI 0.0–54) of cases for *C. coli* and eating chicken pâté was attributed to 6.0% (95% CI 0.0–11).

**Conclusions:**

The main risk factor for campylobacteriosis in Australia is consumption of chicken meat. However, contact with young pet dogs may also be an important source of infection. Proton-pump inhibitors are likely to increase vulnerability to infection.

**Supplementary Information:**

The online version contains supplementary material available at 10.1186/s12879-022-07553-6.

## Background

*Campylobacter* spp. are among the most common bacterial causes of diarrheal disease worldwide [1, 2]. In Australia, the National Notifiable Diseases Surveillance System (NNDSS) has recorded data on campylobacteriosis in seven of eight jurisdictions since 1991. Over this period, the notification rate has generally increased. Campylobacteriosis was mandated as notifiable in all jurisdictions from 2017 [3]. In 2019, Australia reported a notification rate of 143.5 cases per 100,000 population [4], higher than recently reported rates per 100,000 population from other high-income countries such as the United States (19.5 cases in 2018) and the United Kingdom (96.8 cases in 2017) [5, 6]. There is considerable underreporting of campylobacteriosis to surveillance systems, with an estimated ten community cases for each reported case in Australia [7].

A large proportion of *Campylobacter* spp. isolates from notified cases in Australia are not speciated. Of the 17 *Campylobacter* species identified as human pathogens, the two most common are *Campylobacter jejuni* (*C. jejuni*) and *Campylobacter coli* (*C. coli*)*,* which are predominantly acquired from animal sources [1, 8]. *Campylobacter* spp. comprise part of the normal gastrointestinal tract flora of poultry, adult ruminants, and other wild and domestic animals such as rodents, birds, and dogs [1, 2, 9]. Infections are often acquired through consumption of undercooked meats (e.g., poultry) or contact with infected animals [1, 10, 11]. While most *Campylobacter* infections are sporadic, outbreaks can occur and are often associated with chicken or chicken-containing dishes, contaminated water, or raw dairy products [1, 12, 13].

The epidemiology of campylobacteriosis is similar across high-income countries, although variations in risk factors may indicate differences in primary sources, human behaviour, and *Campylobacter* ecology. Such variations include stronger associations with zoonotic factors (e.g., contact with animal faeces, farm animals, and pet dogs aged less than 6 months), consuming barbecued foods, frequency and prevalence of poultry consumption, consuming bottled water, living on a farm, or contact with environmental sources (e.g., garden soil) depending on study location and design [14–16].

The majority of human campylobacteriosis is caused by *C. jejuni* (> 80%), with nearly all remaining cases caused by *C. coli* [2, 17–19]. Historically, studies have not always determined the species causing campylobacteriosis and thus few risk factors have been attributed specifically to either *C. jejuni* or *C. coli,* although *C. coli* campylobacteriosis cases tend to be older in age [19, 20].

In this paper, we present the findings from the CampySource case–control study investigating risk factors associated with sporadic campylobacteriosis caused by *C. jejuni* and *C. coli* in Australia [21].

## Methods

### Study design and population

Study design, participant recruitment, and data collection followed the CampySource protocol [21]. Data collection for the multijurisdictional case–control study occurred over a period of 20 months from February 2018 through October 2019 in the Australian Capital Territory (ACT), Queensland (Qld), and the Hunter New England (HNE) region of New South Wales, covering a total population of approximately 6.1 million people [21]. Health units from each jurisdiction provided lists of participants to a specialised computer-assisted telephone interviewing (CATI) team conducting interviews with all Qld and HNE participants and ACT controls, and to ACT Health staff conducting interviews of ACT cases.

### Case and control recruitment

Recruitment of cases and controls is described elsewhere [21]. Briefly, people with culture-confirmed campylobacteriosis were eligible for interview if they had recent acute diarrhoea with three or more loose bowel movements in a 24-h period and were able to recall the illness onset date. Cases were excluded if an additional enteric pathogen to *Campylobacter* was detected in their stool. Controls were recruited from among notified cases of influenza with a delay of at least 6 months from their reported illness. Controls were frequency matched to cases by sex, age group, and location. We estimated that a sample size of 1200 participants (600 cases; 600 controls) was necessary to enable the detection of statistically significant associations for risk factors of interest to a *p*-value of *p* = 0.05, with 80% power and considering a range of magnitudes of odds ratios to be detected and prevalence of exposure amongst the controls, as previously described [21].

Cases and controls were excluded from interview if: (1) a household member was positive for *Campylobacter* or experienced diarrhoea in the 4 weeks prior to illness onset or interview, (2) they travelled outside of Australia during (or interstate for the entire duration of) the 2 weeks prior to illness onset or interview, (3) they could not speak English or were unable to answer questions for another reason, (4) they were unable to be contacted after six telephone attempts, (5) they did not have a telephone number available (residential or mobile), or (6) they resided outside the study catchment areas.

### Questionnaire

Telephone-administered questionnaires were used to collect information on known risk factors for campylobacteriosis in the 7 days prior to case illness onset, and in the 7 days prior to interview for controls. The questionnaire investigated clinical features, demographic details, and various potential risk factors, as described previously [21].

### Isolate speciation

Stool samples from potential cases were collected and *Campylobacter* spp. isolated [21]. Genome sequencing of isolates from patients included in the study was subsequently conducted using the Illumina^®^ sequencing platform [22]. Taxonomic classification to species-level for each isolate was determined from isolate read sets using Kraken with the PlusPf database [23]. Primary genome sequencing data for each isolate were submitted to the National Centre for Biotechnology Information (NCBI) and are included in Bioprojects PRJNA592186 and PRJNA560409. A total of 63 study isolates could not be revived in culture and classification was not confirmed beyond genus level.

## Data analysis

Data cleaning, variable manipulation, and statistical analyses were performed with R (version 4.0.5) [24], using the ‘dplyr’ package (version 1.0.5) to clean and manipulate variables [25], ‘arsenal’ (version 3.6.2) to create summary tables and perform univariable logistic regressions [26], ‘stats’ (version 4.0.5) for multivariable regressions [27], ‘questionr’ (version 0.7.4) to produce odds ratios [28], ‘rcompanion’ (version 2.4.1) for Cramér’s V [29], and ‘rms’ (version 6.2-0) for variance inflation factor estimation (VIF) [30]. Logistic regression modelling was used to calculate adjusted odds ratios controlling for study design variables of age group, sex, location, and season for every variable in the dataset. A *p*-value threshold of *p* ≤ 0.10 was used to determine variable inclusion for further logistic regression modelling in six separate exposure groups: (1) demographics, illnesses, and medications; (2) water consumption; (3) food (excluding poultry); (4) poultry; (5) animals and pets; and (6) food preparation.

Within each exposure group, we used backward stepwise logistic regression to identify variables to be considered for inclusion in the combined multivariable model, including those with *p-*values ≤ 0.05 and variables with *p*-values between *p* = 0.05 and *p* = 0.1 that were plausible risk factors. Following this, we assessed the VIF of each exposure group, adopting the commonly used cut-off value of ≥ 5 to identify variable collinearity [31]. Pairs of high-VIF variables were then assessed using Cramér’s V and a cut-off guide of V > 0.25 [32] to determine which variables were driving collinearity, keeping the most biologically plausible variables in the model (Additional file 1). Three final models were created with the outcome variable based on species of infection: (1) *Campylobacter* spp., (2) *C. jejuni*, and (3) *C. coli.*

We performed a sensitivity analysis on the *Campylobacter* spp. model by including only those participants that ate chicken to investigate any differences in risk factors between the whole population and those who consumed chicken meat using the same methodology as above. Due to lack of power related to insufficient sample population sizes, we did not perform this sensitivity analysis separately for the *C. jejuni* and *C. coli* specific models.

We calculated the population attributable fraction (PAF) for elevated risk factors for all campylobacteriosis cases and those caused by *C. jejuni* or *C. coli* using the R package ‘AF’ (version 0.1.5) [33], with 95% bootstrap confidence intervals calculated using the R package ‘boot’ [34]. We define the PAF as the fraction of cases (from all species, *C. jejuni*, or *C. coli*) in the population that would be averted if a risk factor was removed from the entire population.

### Ethics

This study was approved by the Australian National University Human Research Ethics Committee (Reference No. 2016/426), ACT Health Human Research Ethics Committee (Reference No. ETH.8.17.168), Qld Health Human Research Ethics Committee (Reference No. RD007108), HNE Human Research Ethics Committee (Reference No. 17/08/16/4.03), and the University of Melbourne Office of Research Ethics and Integrity (Reference No. 1750366.1). Human ethics approval was obtained for patients with campylobacteriosis in the ACT, HNE, and Qld as part of the CampySource case–control study [21].

## Results

We invited 939 cases and 1,988 controls to participate in the study. The study team completed interviews for 571 cases (61% response rate) and 586 controls (29% response rate). The median delay from onset of illness in cases to interview was 19 days (range: 3–66 days). Of the cases, 422 (74%) were infected with *C. jejuni,* 84 (15%) with *C. coli,* two with *C. lari* (< 1%), and 63 (11%) were not identified due to inability to recover isolates in culture. One case was infected with both *C. jejuni* and *C. coli* and was included in the individual analyses for each species.

The distribution of age and sex for study cases was consistent with those notified in Australia 2018–2019 (Additional file 2) with no statistically significant differences between cases and controls for any of the demographic factors assessed. *C. coli* cases were older than *C. jejuni* cases (mean age 44.4 years versus 38.1 years, respectively, *p* = 0.018), and a higher proportion of *C. coli* cases were males compared to *C. jejuni* cases, although this wasn’t statistically significant (64% versus 56%, respectively, *p* = 0.196) (Table 1).

**Table 1:** Demographic characteristics of the CampySource Project case–control study for all campylobacteriosis cases, *C. jejuni* cases, *C. coli* cases, and controls, Australia, 2018–2019

Characteristic		All cases (*n* = 571) *n* (%)	Controls (*n* = 586) *n* (%)	*C. jejuni* cases (*n* = 422) *n* (%)	*C. coli* cases (*n* = 84) *n* (%)	*P* value*
Sex	Female	241 (42)	244 (42)	186 (44)	30 (36)	0.196
	Male	330 (58)	342 (58)	236 (56)	54 (64)	
Jurisdiction	ACT	93 (16)	93 (16)	64 (15)	5 (6)	0.076
	HNE	178 (31)	192 (33)	116 (28)	27 (32)	
	Qld	300 (53)	301 (51)	242 (57)	52 (62)	
Age	Mean (*SD*)	39.4 (24.0)	40.2 (23.9)	38.1 (24.1)	44.4 (21.9)	0.018
Rurality	Rural or remote	81 (14)	75 (13)	66 (16)	9 (11)	0.317
Aboriginal and Torres Strait Islander status	Aboriginal and Torres Strait Islander person	28 (5)	27 (5)	22 (5)	3 (4)	0.783
Season	Summer	145 (25)	139 (24)	102 (24)	28 (33)	0.354
	Autumn	147 (26)	120 (21)	112 (27)	18 (21)	
	Winter	159 (28)	204 (35)	118 (28)	22 (26)	
	Spring	120 (21)	119 (20)	90 (21)	16 (19)	
Highest level of education	Year 10	66 (12)	64 (11)	46 (11)	13 (15)	0.312
	Year 12	77 (14)	75 (13)	62 (15)	6 (7)	
	TAFE/apprenticeship	145 (25)	151 (26)	106 (25)	19 (23)	
	Undergraduate degree	160 (28)	157 (27)	122 (29)	25 (30)	
	Postgraduate degree	114 (20)	134 (23)	79 (19)	19 (23)	
	Unknown or refused	8 (1)	3 (< 1)	6 (1)	2 (2)	
Household income in AUD per annum	< $25 k	35 (6)	39 (7)	24 (6)	7 (8)	0.874
	$25–50 k	93 (17)	81 (14)	67 (16)	14 (17)	
	$50–100 k	145 (25)	124 (21)	113 (27)	20 (24)	
	$100–150 k	122 (21)	146 (25)	85 (20)	17 (20)	
	> $150 k	118 (21)	140 (24)	87 (20)	19 (23)	
	Unknown or refused	57 (10)	54 (9)	45 (11)	7 (8)	
Language in household	Language other than English	52 (9)	52 (9)	36 (9)	7 (8)	1.000

*ACT* Australian Capital Territory, *HNE* Hunter New England region of New South Wales, *Qld* Queensland, *TAFE* Technical and Further Education

*t test, Fisher’s test, or Pearson’s Chi-squared test of the difference between *C. jejuni* and *C. coli* cases

Univariate analyses for all risk factors for *Campylobacter* spp., *C. jejuni,* and *C. coli* variables adjusted for age, sex, location, and season are presented in Additional files 3, 4 and 5. These include meat consumption frequency and food preference variables we excluded from the main models (i.e., “number of chicken meals consumed” and “number of days in 7 days prior to illness that meat was consumed”) as they did not show a dose–response relationship and were collinear with other variables. The final multivariable logistic regression models for *Campylobacter* spp., *C. jejuni,* and *C. coli* are shown in Table 2.

**Table 2:** Results of multivariable logistic regression analysis showing adjusted odds ratios (aOR) and 95% confidence intervals (95% CI) for exposures associated with an increased or decreased campylobacteriosis risk, Australia, 2018–2019

Exposures	*Campylobacter* aOR (95% CI) *n* = 1006	*C. jejuni* aOR (95% CI) *n* = 899	*C. coli* aOR (95% CI) *n* = 638
Medication exposures in 4 weeks prior to illness			
Antibiotics	0.4 (0.2–0.7)	0.3 (0.2–0.6)	0.3 (0.0–0.9)
Proton-pump inhibitors	2.8 (1.9–4.3)	2.6 (1.7–4.2)	3.8 (1.9–7.8)
Poultry-related food exposures in 7 days prior to illness			
Ate duck	3.1 (1.1–9.7)	–	–
Ate pre-cooked chicken	–	–	0.4 (0.2–0.9)
Ate cooked chicken kebabs	2.2 (1.4–3.8)	2.0 (1.1–3.5)	–
Chicken consumption			
None	Ref.	Ref.	Ref.
Ate cooked chicken only	1.7 (1.1–2.8)	1.7 (1.0–3.0)	2.0 (0.8–6.1)
Ate undercooked chicken	70 (13–1296)	85 (15–1627)	60 (6.6–1408)
Other food exposures in 7 days prior to illness			
Ate at a café or restaurant	0.7 (0.5–1.0)	–	–
Ate at a fast food or takeaway outlet*	0.6 (0.5–0.9)	–	–
Ate delicatessen ham, chicken, turkey or beef	–	–	1.8 (1.0–3.3)
Ate chicken pâté	–	–	6.1 (1.5–25)
Ate minced beef or veal dishes	0.7 (0.5–0.9)	–	–
Beef consumption			
None	–	Ref.	–
Ate cooked beef only	–	0.8 (0.5–1.1)	–
Ate undercooked beef	–	0.3 (0.1–0.7)	–
Lamb consumption			
None	Ref.	Ref.	–
Ate cooked lamb only	0.6 (0.4–0.8)	0.6 (0.5–0.9)	–
Ate undercooked lamb	0.2 (0.0–0.9)	0.4 (0.0–1.8)	–
Pork consumption			
None	–	Ref.	–
Ate cooked pork only	–	0.7 (0.5–0.9)	–
Ate undercooked pork	–	3.6 (0.1–97)	–
Animal exposures			
Contact with chicken faeces in 7 days prior to illness	4.3 (1.7–13)	4.3 (1.5–14)	–
Visited a private farm in 7 days prior to illness	–	2.1 (1.1–3.7)	–
Fed pet dog raw chicken necks	–	1.8 (1.0–3.2)	–
Age of pet cat			
No cat	–	Ref.	–
Cat aged less than 6 months	–	3.8 (1.2–13)	–
Cat aged more than 6 months	–	1.0 (0.7–1.4)	–
Age of pet dog			
No dog	Ref.	Ref.	–
Dog aged less than 6 months	6.4 (3.4–12)	6.2 (3.2–12)	–
Dog aged more than 6 months	1.3 (1.0–1.7)	1.2 (0.8–1.6)	–
Preparation exposures in 3 months prior to illness			
Cooked meat on barbecue	–	–	0.5 (0.3–0.8)

*Excludes kebab shops. ‘–’ variable was not included in model

Use of PPIs in the 4 weeks prior to illness was significantly associated with campylobacteriosis (aOR 2.8, 95% CI 1.9–4.3). Taking antibiotics in the 4 weeks prior to illness was associated with reduced odds of campylobacteriosis (aOR 0.4, 95% CI 0.2–0.7) (Table 2). These factors remained significant when analysed separately for *C. jejuni* or *C. coli* infections*.*

Eating cooked chicken (aOR 1.7, 95% CI 1.1–2.8) or undercooked chicken (aOR 70, 95% CI 13–1296) were strongly statistically associated with campylobacteriosis. The wide confidence interval for undercooked chicken was due to few study participants (37 cases, 1 control) reporting this exposure (Additional file 3). Eating undercooked chicken remained significantly associated with illness when analysed separately for *C. jejuni* and *C. coli* infections. However, eating cooked chicken was not statistically significantly associated with *C. coli* infection (aOR 2.0, 95% CI 0.8–6.1). Eating chicken kebabs (aOR 2.2, 95% CI 1.4–3.8) and duck (aOR 3.1, 95% CI 1.1–9.7) were associated with campylobacteriosis. Eating chicken kebabs remained a risk factor for *C. jejuni* (aOR 2.0, 95% CI 1.1–3.5), but was not associated with *C. coli* infection*.* Eating pre-cooked chicken was associated with reduced odds of *C. coli* infection (aOR 0.4, 95% CI 0.2–0.9). Eating chicken pâté (aOR 6.1, 95% CI 1.5–25) and delicatessen ham, chicken, turkey, or beef (aOR 1.8, 95% CI 1.0–3.3), also known as cold cuts or sliced meats, were significantly associated with *C. coli* infection.

Some foods and food exposure locations were associated with reduced odds of campylobacteriosis. These included cooking food on a barbecue, eating at locations outside of the home (e.g., café or fast-food outlet), and consumption of non-poultry meats such as minced beef or veal and cooked lamb (Table 2).

Contact with chicken faeces (aOR 4.3, 95% CI 1.7–13) or owning a pet dog aged less than 6 months (aOR 6.4, 95% CI 3.4–12) were associated with campylobacteriosis. These factors remained significant for *C. jejuni* cases, with the addition of owning a pet cat aged less than 6 months (aOR 3.8, 95% CI 1.2–13), visiting a private farm (aOR 2.1, 95% CI 1.1–3.7), and feeding a pet dog raw chicken necks (aOR 1.8, 95% CI 1.0–3.2). No animal exposures were significantly associated with illness from *C. coli* infection.

We estimated that 42% (95% CI 13–62) of campylobacteriosis cases in the study population were attributable to any chicken consumption (cooked or undercooked), while PPI use was attributed to 13% (95% CI 8.3–18) of cases and owning a pet dog aged less than 6 months was attributable to 9.6% (95% CI 6.5–13) of cases (Table 3). The PAF for the other independent risk factors ranged from 1.8 to 7.0%. The PAF for these variables remained similar for *C. jejuni* and *C. coli* infections. The animal variables associated with only *C. jejuni* infection ranged from 2.4 to 7.0%. Eating delicatessen meats was attributed to 31% (95% CI 0.0–54) of cases for *C. coli* infection and eating chicken pâté was attributed to 6.0% (95% CI 0.0–11).

**Table 3:** Population attributable fraction (PAF) proportions with 95% confidence intervals (95% CI) for exposures associated with increased campylobacteriosis risk, Australia, 2018–2019*

Exposures	*Campylobacter* PAF % (95% CI)	*C. jejuni* PAF % (95% CI)	*C. coli* PAF % (95% CI)
Medication exposures			
Proton-pump inhibitors	13 (8.3–18)	12 (6.3–16)	20 (7.8–32)
Poultry-related food exposures			
Ate any chicken^b^	42 (13–62)	42 (11–66)	^a^
Ate cooked chicken	36 (7.5–58)	36 (4.0–60)	^a^
Ate undercooked chicken	6.8 (4.5–9.5)	7.0 (4.9–10)	7.0 (2.2–15)
Ate cooked chicken kebabs	7.0 (3.1–11)	5.7 (1.3–10)	–
Ate duck	1.8 (0.1–3.7)	–	–
Food exposures			
Ate delicatessen ham, chicken, turkey, or beef	–	–	31 (0.0–54)
Ate chicken pâté	–	–	6.0 (0.0–11)
Animal exposures			
Dog aged less than 6 months	9.6 (6.5–13)	11 (6.9–14)	–
Visited a private farm in 7 days prior to illness	–	4.7 (0.4–7.9)	–
Fed pet dog raw chicken necks	–	4.6 (0.4–8.5)	–
Contact with chicken faeces in 7 days prior to illness	3.1 (1.2–5.2)	2.9 (0.8–5.1)	–
Cat aged less than 6 months	–	2.4 (0.3–4.5)	–

*Calculated from adjusted odds ratios (aOR) from final multivariable logistic regression model ‘–’ variable was not included in model. ^a^Variable not significant. ^b^Cooked and undercooked chicken combined

A sensitivity analysis including only participants who ate chicken in the 7 days prior to illness did not indicate any further significant risk factors (Additional file 6).

## Discussion

In the present study, we found the main risk factors for campylobacteriosis in Australia are consumption of chicken meat, PPI use, and contact with young dogs aged less than 6 months. This is consistent with previous case–control studies of sporadic campylobacteriosis in Australia and New Zealand [11]. While undercooked chicken consumption poses a substantial campylobacteriosis risk, cooked chicken consumption has a population attributable risk more than five times higher, accounting for about a third of the campylobacteriosis cases in the population. We identified that these main risk factors were generally consistent across the two most common *Campylobacter* species implicated in human illness, *C. jejuni* and *C. coli*. However, *C. coli* infections were not significantly associated with animal exposures, and unique factors included consumption of chicken pâté and some delicatessen meats (ham, chicken, turkey, or beef).

Differences in case profiles and risk factors between *C. jejuni* and *C. coli* have been reported previously. Two studies reported that cases infected with *C. coli* tended to be older and more likely to have recently travelled abroad [19, 20] and that *C. jejuni* infection is more prevalent in summer [19, 20]. Previously reported principal risk factors for *C. coli* infection include consumption of halal meats, offal meats, pâté, game meat, and market stall foods, while contact with animals has been less commonly reported as a risk factor [20, 35, 36]. Our study found the main risk factors for *C. jejuni* appeared similar to those for campylobacteriosis in general, as reported elsewhere [16]. *C. jejuni* and *C. coli* infections share the risk factors of chicken and other poultry consumption, undercooked meat, and PPI use [35].

Poultry is a well-known reservoir of *Campylobacter* spp. with the pathogen frequently isolated from chicken faeces [37, 38]. Human campylobacteriosis is often attributed to consumption of chicken meat, with Australian and New Zealand case–control studies attributing almost 30% and 5–16% of campylobacteriosis cases to chicken consumption factors, respectively [10, 36]. In particular, raw and undercooked chicken meat are commonly known food vehicles for gastroenteritis [39, 40]. The risk associated with eating cooked chicken is less clear but may be explained through surface or utensil cross-contamination from raw chicken during meal preparation, or cases being unaware they consumed undercooked chicken [10]. Pre-cooked retail chicken consumption was negatively associated with *C. coli* infection. This is likely due to thermal inactivation of *Campylobacter* spp. and reduced likelihood of cross-contamination during meal preparation. Pâté is a known source of *Campylobacter* and is often associated with disease outbreaks [41–44]. Chicken livers are a common retail pâté ingredient and are known to contain *Campylobacter* [45, 46], with recent testing of chicken offal in Australia finding *C. coli* significantly more common in this source than *C. jejuni* [46].

Previous case–control studies have identified PPI use as a risk factor contributing to human *Campylobacter* infection [35, 36, 47, 48]. Increases in campylobacteriosis incidence may also be associated with increased PPI use [49]. PPIs reduce stomach acid secretion and neutralise stomach pH levels [50, 51], and are commonly prescribed to treat acid-related gastro-oesophageal disorders and prevent ulcers [50–52]. Although *Campylobacter* is acid-sensitive, the protective effect of food paired with PPI use could enhance survival of the pathogen through the stomach and into the bowel [53, 54]. While considered safe and effective medications approved for long-term use, they are associated with an increased risk of enteric infection [51, 54]. Many patients use PPIs as a first-line therapy and continue using them on a long-term basis [52]. Additionally, over-prescription of PPIs is a known problem with between 25 and 70% of users lacking an appropriate indicator for use [55]. PPIs are advertised directly to consumers in Australia, and are available for purchase over the counter [52].

Odds of human campylobacteriosis were lower in participants who reported taking antibiotics in the 4 weeks prior to interview. A recent Australian study showed low levels of antimicrobial resistance in campylobacteriosis case isolates relative to other countries [22]. It is plausible that antibiotic use could have a protective effect against campylobacteriosis, with a Danish study finding macrolides provided protection for 4–8 weeks after use [56]. Macrolides are prescribed in Australia for respiratory tract infections, so may have been taken by study controls following influenza infection [57]. However, those that used antibiotics in the 4 weeks prior to interview generally reported using amoxicillin or cephalexin, the most frequently dispensed antimicrobials in Australia [58]. A protective effect has not previously been reported for these antimicrobials.

Exposure to domestic pets has been reported as a risk factor for human campylobacteriosis [10, 47, 59–63], with up to 25% of human campylobacteriosis cases attributed to pet sources [64–66]. Domestic cats and dogs are often asymptomatic carriers of *Campylobacter* spp. [62]. *C. jejuni* is the most commonly isolated *Campylobacter* species in dogs, accounting for over 96% of dog-sourced isolates on PubMLST [67–69]. We found strong associations between owning pet dogs aged less than 6 months and pet cats aged less than 6 months (when restricted to *C. jejuni* infection) and campylobacteriosis. Younger pet dogs are more likely to be a source of infection for human campylobacteriosis as they often have a higher carriage prevalence of *Campylobacter* compared with mature pet dogs (76% in puppies versus 39% in mature dogs), and are more likely to shed *Campylobacter* than older dogs [70]. Newly acquired puppies and kittens present the highest risk of transmission [62].

Raw meat-based diets in pet dogs pose a higher risk of *Campylobacter* spp. carriage than commercial dry feed diets [71]. These diets for domestic pets have increased in popularity, with health benefits including coat and skin improvements and dental diseases reduction [68, 69]. However, raw meats often contain enteric pathogens that may cause serious health conditions in dogs and can be transmitted to humans [68, 72].

Additionally, a high magnitude association was observed for owners that fed their cat raw kangaroo meat (OR 9, 95% CI 1.4–177) and *C. jejuni* infection, but due to small numbers the effect was not statistically significant in multivariable modelling. In Australia, veterinarians may advise feeding cats with severe food allergies raw kangaroo meat as it has markedly different allergenic properties to other commonly available meats. If this raw kangaroo meat has high levels of *Campylobacter* spp. then freezing for at least 1 week [73] and thawing just before feeding could reduce bacterial counts and risk in the owners and their pets.

### Study limitations

The large study size and representativeness of the case population are important strengths of our study. However, our study is subject to biases common to many case–control studies. We had low power for *C. coli* analyses due to a smaller sample population (n = 84), which may have impacted our ability to detect associations. Influenza cases were recruited as controls for their similar healthcare-seeking behaviours. However, there were differences in case and control recruitment rates, with 39% of eligible controls declining interview compared with 9% of eligible cases.

Most controls were interviewed within a 30-day window of cases to ensure minimal variation in seasonal food intake. However, due to difficulties in control recruitment, it was necessary to recruit some controls outside of this window to achieve the 1:1 case:control ratio. We accounted for this by controlling for season throughout all analyses. Interviewers were thoroughly trained and followed interview protocols. However, interviews were not conducted blindly so interviewer bias cannot be ruled out.

The study was designed to minimise information bias by strict recall periods for cases (7 days prior to illness) and controls (7 days prior to interview), and strict adherence to exclusion criteria. However, on average 3 weeks elapsed between illness and interview for cases, which may have resulted in poorer recall and bias toward the null [74]. Cases may have closely reflected upon their food consumption in the days prior to falling ill, explaining some of the difference in responses between cases and controls for factors like undercooked chicken consumption. However, a previous study found no measurable difference in dietary recall between those with and without gastroenteritis symptoms [75], suggesting this has minimal impact on participant responses.

## Conclusions

Australian retail raw meat (including poultry) is not subject to specified microbiological limits [76]. However, voluntary guidelines of less than 6000–10,000 Colony Forming Units (CFU) of *Campylobacter* spp. per poultry carcass exist for the Australian poultry sector [77]. Importantly, doses as low as 360–800 CFU can result in campylobacteriosis [78, 79]. As *Campylobacter* spp. are generally incapable of ex vivo growth in foods, the principal risks associated with campylobacteriosis and chicken meat are undercooking and/or cross-contamination during food preparation. This is particularly the case in relation to chicken liver pâté given the relative prevalence of *Campylobacter* spp. in chicken offal [46, 80, 81].

Australia has a high incidence of human campylobacteriosis compared with other high-income countries, a high prevalence of *Campylobacter* spp. on raw retail poultry [46], and a high population-level campylobacteriosis risk from chicken meat consumption. Given this, communication with, and improved education of, consumers regarding the risks associated with handling raw meats (particularly poultry) including proper food handling, preparation and hygiene practices is recommended as a key approach for personal risk reduction [82, 83]. Additionally, continued engagement with industry partners, particularly in the poultry supply chain, is required to identify means of reducing *Campylobacter* prevalence in, and concentration of *Campylobacter* on, chicken meat.

While clear benefits exist for the use of PPIs, consumers and clinicians should consider these against the risks associated with use before initiating and continuing PPI therapy. PPIs should come with advice that they may increase the risk of gastroenteritis from *Campylobacter* infection and other pathogens including *Salmonella* and *Clostridiodes difficile*.

To reduce the risk of *Campylobacter* spp. transmission from pets to humans, several activities are recommended. These include good hand hygiene practices following animal handling; feeding young pets cooked meat products or commercial canned and dry foods; routine cleaning and disinfection of animal contact surfaces in the home, enclosures, breeding locations, animal shelters, and pet stores; and closely monitoring pet health by engaging with veterinarians for preventative and diagnostic care [62].

We observed differences in risk factors between *C. jejuni* and *C. coli* infections, suggesting that important differences in infection sources may exist between these two species. Future studies should consider high-powered disaggregated *Campylobacter* spp. analyses to detect possible species-specific risk factors. This study provided insights into the public health importance of sources of *Campylobacter* infection and reiterated that awareness of the risks associated with chicken meat handling, preparation, and consumption; PPI use; and contact with young pets is essential for informed control over risk exposure in individuals.

## Supplementary Information

**Additional file 1:** Process for logistic regression model selection for all campylobacteriosis, *Campylobacter jejuni*, and *Campylobacter coli* sensitivity analysis.

**Additional file 2:** Age and sex distribution of study cases (n = 571) compared to notified cases of campylobacteriosis in Australia in 2018 (n = 33,129) and 2019 (n = 36,131)*.

**Additional file 3:** Univariable results for campylobacteriosis, adjusted for age group, sex, location and season.

**Additional file 4:** Univariable results for *Campylobacter jejuni*, adjusted for age group, sex, location and season.

**Additional file 5:** Univariable results for *Campylobacter coli*, adjusted for age group, sex, location and season.

**Additional file 6:** Sensitivity analysis of final multivariable model for participants that consumed chicken with adjusted odds ratios (aORs) together with 95% confidence intervals (95% CI) showing exposures associated with an increased or decreased risk of campylobacteriosis.

## Data Availability

The datasets generated and/or analysed during the current study are available in the National Centre for Biotechnology Information (NCBI) repository, under Bioproject accession numbers PRJNA592186 (https://www.ncbi.nlm.nih.gov/bioproject/592186) and PRJNA560409 (https://www.ncbi.nlm.nih.gov/bioproject/560409).

## Abbreviations

ACT: Australian Capital Territory
aOR: Adjusted odds ratio
*C. coli*: *Campylobacter coli*
*C. jejuni*: *Campylobacter jejuni*
CATI: Computer-assisted telephone interview
CFU: Colony forming units
CI: Confidence interval
HNE: Hunter New England region of New South Wales
NCBI: National Center for Biotechnology Information
NNDSS: National Notifiable Diseases Surveillance System
PAF: Population attributable fraction
PPI: Proton-pump inhibitor
Qld: Queensland
TAFE: Technical and further education
VIF: Variance inflation factor

## References

[1] Kaakoush NO, Castaño-Rodríguez N, Mitchell HM, Man SM (2015). Global epidemiology of *Campylobacter* infection. Clin Microbiol Rev.

[2] Fitzgerald C (2015). *Campylobacter*. Clin Lab Med.

[3] Communicable Diseases Branch. NSW OzFoodNet Annual Surveillance Report: 2018. Sydney; 2019.

[4] Australian Government Department of Health. National Notifiable Diseases Surveillance System—Notification rate of campylobacteriosis 2019 [cited Dec 2021]. Available from: http://www9.health.gov.au/cda/source/cda-index.cfm.

[5] Tack DM, Marder EP, Griffin PM, Cieslak PR, Dunn J, Hurd S (2019). Preliminary incidence and trends of infections with pathogens transmitted commonly through food—foodborne diseases active surveillance network, 10 U.S. Sites, 2015–2018. MMWR Morb Mortal Wkly Rep.

[6] Public Health England. Zoonoses Report UK 2017. London; 2018.

[7] Hall G, Yohannes K, Raupach J, Becker N, Kirk M (2008). Estimating community incidence of *Salmonella, Campylobacter*, and Shiga toxin-producing *Escherichia coli* infections, Australia. Emerg Infect Dis.

[8] Butzler J-P (2004). *Campylobacter*, from obscurity to celebrity. Clin Microbiol Infect.

[9] Inglis GD, Kalischuk LD, Busz HW, Kastelic JP (2005). Colonization of cattle intestines by *Campylobacter jejuni* and *Campylobacter lanienae*. Appl Environ Microbiol.

[10] Stafford RJ, Schluter P, Kirk M, Wilson A, Unicomb L, Ashbolt R (2007). A multi-centre prospective case-control study of campylobacter infection in persons aged 5 years and older in Australia. Epidemiol Infect.

[11] Varrone L, Glass K, Stafford RJ, Kirk MD, Selvey L (2020). A meta-analysis of case-control studies examining sporadic campylobacteriosis in Australia and New Zealand from 1990 to 2016. Aust N Z J Public Health.

[12] Gilpin BJ, Walker T, Paine S, Sherwood J, Mackereth G, Wood T (2020). A large scale waterborne campylobacteriosis outbreak, Havelock North. New Zealand J Infect.

[13] Moffatt CRM, Fearnley E, Bell R, Wright R, Gregory J, Sloan-Gardner T (2019). Characteristics of *Campylobacter* gastroenteritis outbreaks in Australia, 2001 to 2016. Foodborne Pathog Dis.

[14] Friedman CR, Hoekstra RM, Samuel M, Marcus R, Bender J, Shiferaw B (2004). Risk factors for sporadic *Campylobacter* infection in the United States: a case-control study in FoodNet sites. Clin Infect Dis.

[15] MacDonald E, White R, Mexia R, Bruun T, Kapperud G, Lange H (2015). Risk factors for sporadic domestically acquired *Campylobacter* infections in Norway 2010–2011: a national prospective case-control study. PLoS ONE.

[16] Mossong J, Mughini-Gras L, Penny C, Devaux A, Olinger C, Losch S (2016). Human campylobacteriosis in Luxembourg, 2010–2013: a case-control study combined with multilocus sequence typing for source attribution and risk factor analysis. Sci Rep.

[17] Ogden ID, Dallas JF, MacRae M, Rotariu O, Reay KW, Leitch M (2009). *Campylobacter* excreted into the environment by animal sources: prevalence, concentration shed, and host association. Foodborne Pathog Dis.

[18] Ibrahim JN, Eghnatios E, El Roz A, Fardoun T, Ghssein G (2019). Prevalence, antimicrobial resistance and risk factors for campylobacteriosis in Lebanon. J Infect Dev Ctries.

[19] Bessède E, Lehours P, Labadi L, Bakiri S, Mégraud F (2014). Comparison of characteristics of patients infected by *Campylobacter jejuni, Campylobacter coli*, and *Campylobacter fetus*. J Clin Microbiol.

[20] Gillespie IA, O'Brien SJ, Frost JA, Adak GK, Horby P, Swan AV (2002). A case-case comparison of *Campylobacter coli* and *Campylobacter jejuni* infection: a tool for generating hypotheses. Emerg Infect Dis.

[21] Varrone L, Stafford RJ, Lilly K, Selvey L, Glass K, Ford L (2018). Investigating locally relevant risk factors for *Campylobacter* infection in Australia: protocol for a case–control study and genomic analysis. BMJ Open.

[22] Wallace R, Bulach D, McLure A, Varrone L, Jennison A, Valcanis M (2021). Antimicrobial resistance of *Campylobacter* spp. causing human infection in Australia: an international comparison. Microb Drug Resist.

[23] Wood DE, Salzberg SL (2014). Kraken: ultrafast metagenomic sequence classification using exact alignments. Genome Biol.

[24] R Core Team. R: A language and environment for statistical computing. R Foundation for Statistical Computing. Vienna, Austria [cited Dec 2021]. Available from: https://www.R-project.org/.

[25] Wickham H, Francois R, Henry L, Muller K, RStudio. dplyr: A Grammar of Data Manipulation [cited Dec 2021]. Available from: https://cran.r-project.org/web/packages/dplyr/index.html.

[26] Heinzen E, Sinnwell J, Atkinson E, Gunderson T, Dougherty G, Votruba P, et al. arsenal: An Arsenal of ‘R’ Functions for Large-Scale Statistical Summaries [cited Dec 2021]. Available from: https://cran.r-project.org/web/packages/arsenal/index.html.

[27] R Core Team. stats: The R Stats Package 2021 [cited Dec 2021]. Available from: https://www.r-project.org/.

[28] Barnier J, Briatte F, Larmarange J. questionr: Functions to Make Surveys Processing Easier [cited Dec 2021]. Available from: https://cran.r-project.org/web/packages/questionr/index.html.

[29] Mangiafico S. rcompanion: Functions to Support Extension Education Program Evaluation [cited Dec 2021]. Available from: https://cran.r-project.org/web/packages/rcompanion/index.html.

[30] Harrell Jr F. rms: Regression Modeling Strategies [cited Dec 2021]. Available from: https://cran.r-project.org/web/packages/rms/index.html.

[31] Craney TA, Surles JG (2002). Model-dependent variance inflation factor cutoff values. Qual Eng.

[32] Akoglu H (2018). User’s guide to correlation coefficients. Turk J Emerg Med.

[33] Dahlqwist E, Sjolander A. AF: Model-Based Estimation of Confounder-Adjusted Attributable Fractions [cited Dec 2021]. Available from: https://cran.r-project.org/web/packages/AF/index.html.

[34] Canty A, Ripley B. boot: Bootstrap Functions (Originally by Angelo Canty for S) [cited Dec 2021]. Available from: https://cran.r-project.org/web/packages/boot/index.html.

[35] Doorduyn Y, Van Den Brandhof WE, Van Duynhoven YTHP, Breukink BJ, Wagenaar JA, van Pelt W (2010). Risk factors for indigenous *Campylobacter jejuni* and *Campylobacter coli* infections in The Netherlands: a case-control study. Epidemiol Infect.

[36] Lake RJ, Campbell DM, Hathaway SC, Ashmore E, Cressey PJ, Horn BJ (2021). Source attributed case-control study of campylobacteriosis in New Zealand. Int J Infect Dis.

[37] Ono K, Yamamoto K (1999). Contamination of meat with *Campylobacter jejuni* in Saitama, Japan. Int J Food Microbiol.

[38] Rasmussen HN, Olsen JE, Jørgensen K, Rasmussen OF (1996). Detection of *Campylobacter jejuni* and *Camp. coli* in chicken faecal samples by PCR. Lett Appl Microbiol.

[39] Bolton FJ, Sails AD, Fox AJ, Wareing DRA, Greenway DLA (2002). Detection of *Campylobacter jejuni* and *Campylobacter coli* in foods by enrichment culture and polymerase chain reaction enzyme-linked immunosorbent assay. J Food Prot.

[40] Kramer JM, Frost JA, Bolton FJ, Wareing DR (2000). Campylobacter contamination of raw meat and poultry at retail sale: identification of multiple types and comparison with isolates from human infection. J Food Prot.

[41] Elson R, Burgess F, Little CL, Mitchell RT, Services tLAC-ooR, Agency tHP (2004). Microbiological examination of ready-to-eat cold sliced meats and pâté from catering and retail premises in the UK. J Appl Microbiol.

[42] Edwards DS, Milne LM, Morrow K, Sheridan P, Verlander NQ, Mulla R (2014). Campylobacteriosis outbreak associated with consumption of undercooked chicken liver pâté in the East of England, September 2011: identification of a dose–response risk. Epidemiol Infect.

[43] Moffatt CRM, Greig A, Valcanis M, Gao W, Seemann T, Howden BP (2016). A large outbreak of *Campylobacter jejuni* infection in a university college caused by chicken liver pâté, Australia, 2013. Epidemiol Infect.

[44] Lahti E, Löfdahl M, Ågren J, Hansson I, Olsson EE (2017). Confirmation of a campylobacteriosis outbreak associated with chicken liver Pâté using PFGE and WGS. Zoonoses Public Health.

[45] Atanassova V, Ring C (1999). Prevalence of *Campylobacter* spp. in poultry and poultry meat in Germany. Int J Food Microbiol.

[46] Walker LJ, Wallace RL, Smith JJ, Graham T, Saputra T, Symes S (2019). Prevalence of *Campylobacter coli* and *jejuni* in retail chicken, beef, lamb and pork products in three Australian states. J Food Prot.

[47] Neal KR, Slack RC (1997). Diabetes mellitus, anti-secretory drugs and other risk factors for campylobacter gastro-enteritis in adults: a case-control study. Epidemiol Infect.

[48] Unicomb LE, O'Reilly LC, Kirk MD, Stafford RJ, Smith HV, Becker NG (2008). Risk factors for infection with *Campylobacter jejuni flaA* genotypes. Epidemiol Infect.

[49] Bouwknegt M, van Pelt W, Kubbinga ME, Weda M, Havelaar AH (2014). Potential association between the recent increase in campylobacteriosis incidence in the Netherlands and proton-pump inhibitor use—an ecological study. Euro Surveill.

[50] Jackson MA, Goodrich JK, Maxan M-E, Freedberg DE, Abrams JA, Poole AC (2016). Proton pump inhibitors alter the composition of the gut microbiota. Gut.

[51] Chen Y, Liu B, Glass K, Du W, Banks E, Kirk M (2016). Use of proton pump inhibitors and the risk of hospitalization for infectious gastroenteritis. PLoS ONE.

[52] Australian Commission on Safety and Quality in Health Care. The Third Australian Atlas of Healthcare Variation 2018—2.3 Proton pump inhibitor medicines dispensing, 18 years and over. 2018.

[53] Lombardo L, Foti M, Ruggia O, Chiecchio A (2010). Increased incidence of small intestinal bacterial overgrowth during proton pump inhibitor therapy. Clin Gastroenterol Hepatol.

[54] Leonard J, Marshall JK, Moayyedi P (2007). Systematic review of the risk of enteric infection in patients taking acid suppression. Am J Gastroenterol.

[55] Forgacs I, Loganayagam A (2008). Overprescribing proton pump inhibitors. BMJ.

[56] Koningstein M, Simonsen J, Helms M, Hald T, Mølbak K (2011). Antimicrobial use: a risk factor or a protective factor for acquiring campylobacteriosis?. Clin Infect Dis.

[57] McMullan BJMM (2015). Prescribing azithromycin. Aust Prescr.

[58] The Australian Commission on Safety and Quality in Health Care. Fourth Australian report on antimicrobial use and resistance in human health. Sydney: ACSQHC; 2021.

[59] Kapperud G, Skjerve E, Bean NH, Ostroff SM, Lassen J (1992). Risk factors for sporadic *Campylobacter* infections: results of a case-control study in southeastern Norway. J Clin Microbiol.

[60] Tenkate TD, Stafford RJ (2001). Risk factors for campylobacter infection in infants and young children: a matched case-control study. Epidemiol Infect.

[61] Moffatt C, Appuhamy R, Andrew W, Wynn S, Roberts J, Kennedy K (2014). An assessment of risk posed by a Campylobacter-positive puppy living in an Australian residential aged-care facility. West Pac Surveill Response J WPSAR.

[62] Stull JW, Brophy J, Weese JS (2015). Reducing the risk of pet-associated zoonotic infections. Can Med Assoc J.

[63] Carrique-Mas J, Andersson Y, Hjertqvist M, Svensson Å, Torner A, Giesecke J (2005). Risk factors for domestic sporadic campylobacteriosis among young children in Sweden. Scand J Infect Dis.

[64] Rosner BM, Schielke A, Didelot X, Kops F, Breidenbach J, Willrich N (2017). A combined case-control and molecular source attribution study of human *Campylobacter* infections in Germany, 2011–2014. Sci Rep.

[65] Mughini-Gras L, Smid JH, Wagenaar JA, Koene MGJ, Havelaar AH, Friesema IHM (2013). Increased risk for *Campylobacter jejuni* and *C. coli* infection of pet origin in dog owners and evidence for genetic association between strains causing infection in humans and their pets. Epidemiol Infect.

[66] Mughini-Gras L, Pijnacker R, Coipan C, Mulder AC, Fernandes Veludo A, de Rijk S (2021). Sources and transmission routes of campylobacteriosis: a combined analysis of genome and exposure data. J Infect.

[67] PubMLST. *Campylobacter jejuni/coli* isolates 2021 [cited Dec 2021]. Available from: https://pubmlst.org/bigsdb?db=pubmlst_campylobacter_isolates&page=query.

[68] Fredriksson-Ahomaa M, Heikkilä T, Pernu N, Kovanen S, Hielm-Björkman A, Kivistö R (2017). Raw meat-based diets in dogs and cats. Vet Sci.

[69] Runesvärd E, Wikström C, Fernström L-L, Hansson I (2020). Presence of pathogenic bacteria in faeces from dogs fed raw meat-based diets or dry kibble. Vet Rec.

[70] Engvall EO, Brändström B, Andersson L, Båverud V, Trowald-Wigh G, Englund L (2003). Isolation and identification of thermophilic *Campylobacter* species in faecal samples from Swedish dogs. Scand J Infect Dis.

[71] Bojanić K, Midwinter AC, Marshall JC, Rogers LE, Biggs PJ, Acke E (2017). Isolation of *Campylobacter* spp. from client-owned dogs and cats, and retail raw meat pet food in the Manawatu, New Zealand. Zoonoses Public Health.

[72] Martinez-Anton L, Marenda M, Firestone SM, Bushell RN, Child G, Hamilton AI (2018). Investigation of the role of campylobacter infection in suspected acute polyradiculoneuritis in dogs. J Vet Intern Med.

[73] Whyte R, Hudson J, Turner N (2005). Effect of low temperature on *Campylobacter* on poultry meat.

[74] Varrone L, Glass K, Stafford RJ, Kirk MD, Selvey L (2020). Validation of questions designed for investigation of gastroenteritis. Food Control.

[75] Seitzinger PJ, Tataryn J, Osgood N, Waldner C (2019). Foodborne outbreak investigation: effect of recall inaccuracies on food histories. J Food Prot.

[76] Food Standards Australia New Zealand. Australia New Zealand Food Standards Code—Standard 2.2.1—Meat and meat products. Canberra: FSANZ; 2015.

[77] Food Standards Australia New Zealand. Compendium of Microbiological Criteria for Food. Canberra, Australia; 2018.

[78] Black RE, Levine MM, Clements ML, Hughes TP, Blaser MJ (1988). Experimental *Campylobacter jejuni* Infection in Humans. J Infect Dis.

[79] Hara-Kudo Y, Takatori K (2011). Contamination level and ingestion dose of foodborne pathogens associated with infections. Epidemiol Infect.

[80] Little CL, Richardson JF, Owen RJ, de Pinna E, Threlfall EJ (2008). *Campylobacter* and *Salmonella* in raw red meats in the United Kingdom: prevalence, characterization and antimicrobial resistance pattern, 2003–2005. Food Microbiol.

[81] New South Wales Food Authority. *Campylobacter* in meat and offal: microbiological quality of beef, lamb and pork meat cuts and offal. 2018.

[82] Allan PD, Palmer C, Chan F, Lyons R, Nicholson O, Rose M (2018). Food safety labelling of chicken to prevent campylobacteriosis: consumer expectations and current practices. BMC Public Health.

[83] Facciolà A, Riso R, Avventuroso E, Visalli G, Delia SA, Laganà P (2017). *Campylobacter:* from microbiology to prevention. J Prev Med Hyg.

