# Supplementary material for: Risk factors for campylobacteriosis in Australia: outcomes of a 2018–2019 case–control study
Source: BMC Infect Dis. 2022 Jun 30;22:586. doi: 10.1186/s12879-022-07553-6 (PMC9245254; doi:10.1186/s12879-022-07553-6)
Supplement: Supplementary file 1 — Additional file 1: Process for logistic regression model selection for all campylobacteriosis, Campylobacter jejuni, and Campylobacter coli sensitivity analysis. [file 12879_2022_7553_MOESM1_ESM.docx]

**Additional file 1.** Process for logistic regression model selection for all campylobacteriosis, *Campylobacter jejuni*, and *Campylobacter coli* sensitivity analysis.


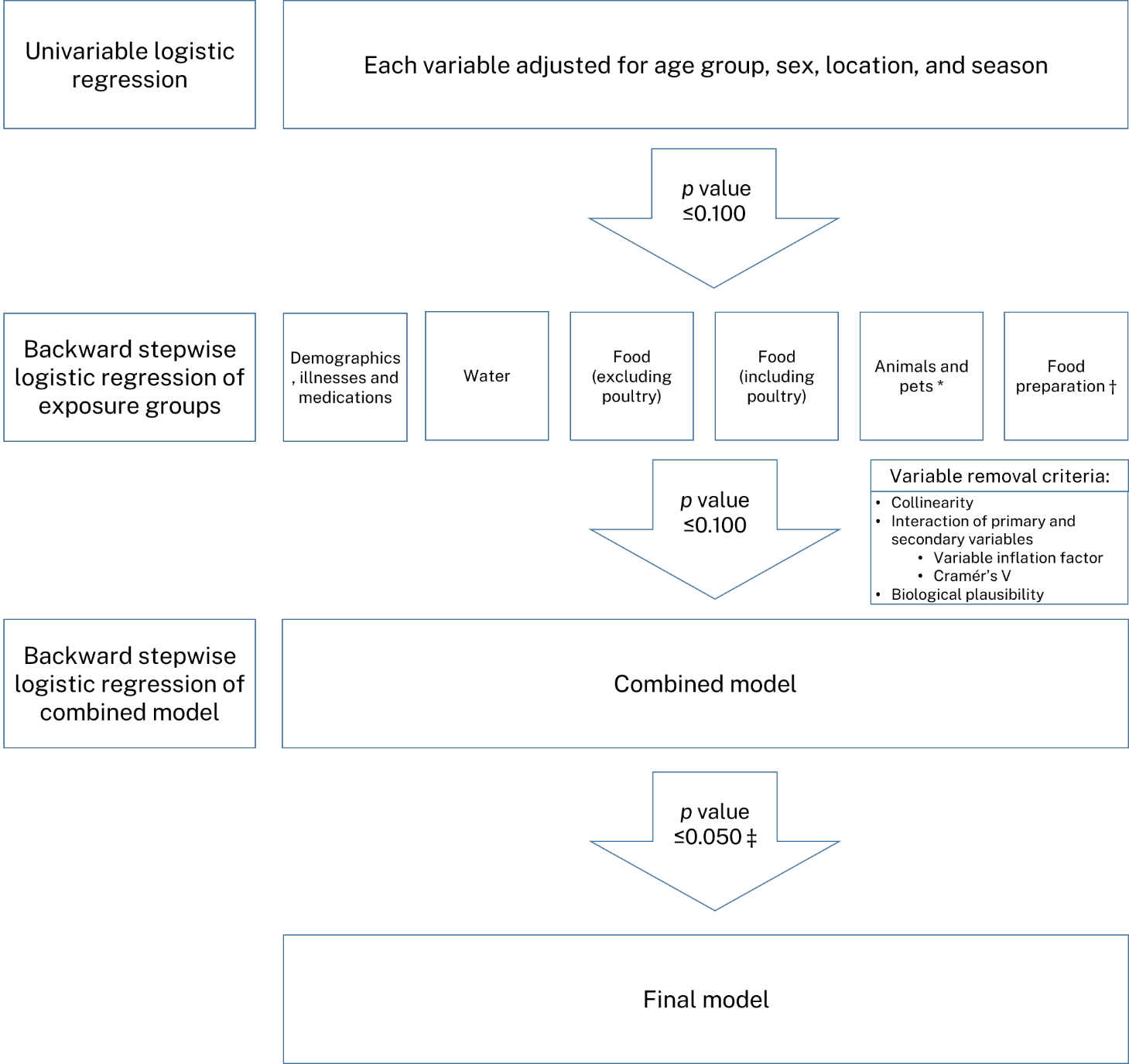


* not used for *Campylobacter coli* model. † not used for all campylobacteriosis model. ‡ *p* value of ≤0.050 or between *p*=0.05 and *p*=0.1 for plausible risk factors that verge on significance.
