# Supplementary material for: Risk factors for campylobacteriosis in Australia: outcomes of a 2018–2019 case–control study
Source: BMC Infect Dis. 2022 Jun 30;22:586. doi: 10.1186/s12879-022-07553-6 (PMC9245254; doi:10.1186/s12879-022-07553-6)
Supplement: Supplementary file 2 — Additional file 2: Age and sex distribution of study cases (n = 571) compared to notified cases of campylobacteriosis in Australia in 2018 (n = 33,129) and 2019 (n = 36,131)*. [file 12879_2022_7553_MOESM2_ESM.docx]

**Additional file 2.** Age and sex distribution of study cases (n=571) compared to notified cases of campylobacteriosis in Australia in 2018 (n=33,129) and 2019 (n=36,131)*.

| **Characteristic** | **Study cases**  **n (%)** | **Notified cases in Australia 2018**  **n (%)^** | **Notified cases in Australia 2019**  **n (%)^** |
| --- | --- | --- | --- |
| **Age (years), p = 0.042**** | | | |
| 0–4 | 49 (8.6) | 3,275 (9.9) | 3,537 (9.8) |
| 5–14 | 66 (11.6) | 2,944 (8.9) | 3,174 (8.8) |
| 15–34 | 144 (25.2) | 9,155 (27.6) | 9,953 (27.5) |
| 35–54 | 131 (22.9) | 7,329 (22.1) | 8,062 (22.3) |
| 55–74 | 141 (24.7) | 7,369 (22.2) | 8,092 (22.4) |
| 75 and over | 40 (7.0) | 3,057 (9.2) | 3,313 (9.2) |
| **Sex, p = 0.230**** | | | |
| Male | 330 (57.8) | 18,220 (55.0) | 19,951 (55.2) |
| Female | 241 (42.2) | 14,849 (44.8) | 16,105 (44.6) |
| Unknown | 0 (0.0) | 74 (0.2) | 76 (0.2) |
| * Totals are from age group counts, which differ from sex (+14 for 2018 and +1 for 2019 for sex).  ^ Data sourced from the National Notifiable Disease Surveillance System (NNDSS).  ** p-value from χ^2^ test of: [study cases] x [([notified 2018] + [notified 2019]) – [study cases]]. | | | |
