# Supplementary material for: Risk factors for campylobacteriosis in Australia: outcomes of a 2018–2019 case–control study
Source: BMC Infect Dis. 2022 Jun 30;22:586. doi: 10.1186/s12879-022-07553-6 (PMC9245254; doi:10.1186/s12879-022-07553-6)
Supplement: Supplementary file 6 — Additional file 6: Sensitivity analysis of final multivariable model for participants that consumed chicken with adjusted odds ratios (aORs) together with 95% confidence intervals (95% CI) showing exposures associated with an increased or decreased risk of campylobacteriosis. [file 12879_2022_7553_MOESM6_ESM.docx]

**Additional file 6.** Sensitivity analysis of final multivariable model for participants that consumed chicken with adjusted odds ratios (aORs) together with 95% confidence intervals (95% CI) showing exposures associated with an increased or decreased risk of campylobacteriosis.

| **Exposures** | **aOR (95% CI)**  **n=910** |
| --- | --- |
| **Medication exposures in 4 weeks prior to illness** | |
| Antibiotics | 0.4 (0.2–0.7) |
| Proton-pump inhibitors | 2.7 (1.8–4.2) |
| **Poultry-related food exposures in 7 days prior to illness** | |
| Ate cooked chicken kebabs | 2.1 (1.3–3.5) |
| Chicken consumption |  |
| Ate cooked chicken only* | ref |
| Ate undercooked chicken | 41 (8.4–750) |
| **Food exposures in 7 days prior to illness** | |
| Ate minced beef or veal dishes | 0.6 (0.4–0.8) |
| Beef consumption |  |
| None | ref |
| Ate cooked beef only | 1.1 (0.7–1.7) |
| Ate undercooked beef | 0.4 (0.2–0.9) |
| Lamb consumption |  |
| None | ref |
| Ate cooked lamb only | 0.6 (0.4–0.8) |
| Ate undercooked lamb | 0.4 (0.1–1.8) |
| Pork consumption |  |
| None | ref |
| Ate cooked pork only | 0.7 (0.5–1.0) |
| Ate undercooked pork | 2.4 (0.2–61) |
| **Animal exposures** | |
| Contact with chicken faeces in 7 days prior to illness | 4.5 (1.8–13) |
| Age of pet dog |  |
| No dog | ref |
| Dog aged less than six months | 6.2 (3.2–13) |
| Dog age more than six months | 1.4 (1.0–1.9) |
| * cooked chicken used as reference group as non-chicken consumers are excluded from analysis | |
